# Circulating tumor DNA sequencing provides comprehensive mutation profiling for pediatric central nervous system tumors

**DOI:** 10.1038/s41698-022-00306-3

**Published:** 2022-09-06

**Authors:** Erin R. Bonner, Robin Harrington, Augustine Eze, Miriam Bornhorst, Cassie N. Kline, Heather Gordish-Dressman, Adam Dawood, Biswajit Das, Li Chen, Rini Pauly, P. Mickey Williams, Chris Karlovich, Amanda Peach, D’andra Howell, James Doroshow, Lindsay Kilburn, Roger J. Packer, Sabine Mueller, Javad Nazarian

**Affiliations:** 1 Center for Genetic Medicine Research, Children’s National Hospital, Washington, DC USA; 2 Institute for Biomedical Sciences, The George Washington University School of Medicine and Health Sciences, Washington, DC USA; 3 Molecular Characterization Laboratory, Frederick National Laboratory for Cancer Research, Frederick, MD USA; 4 Division of Oncology, Department of Pediatrics, Children’s Hospital of Philadelphia, University of Pennsylvania Perelman School of Medicine, Philadelphia, PA USA; 5 Department of Biostatistics, Children’s National Hospital, Washington, DC USA; 6 Division of Cancer Treatment and Diagnosis, Developmental Therapeutics Clinic/Early Clinical Trials Development Program, National Cancer Institute, Bethesda, MD USA; 7 Brain Tumor Institute, Children’s National Hospital, Washington, DC USA; 8 Department of Neurology, Neurosurgery and Pediatrics, University of California San Francisco, San Francisco, CA USA; 9 Department of Pediatrics, University Children’s Hospital Zürich, Zürich, Switzerland

**Keywords:** CNS cancer, Paediatric cancer, Tumour biomarkers, Next-generation sequencing, Next-generation sequencing

## Abstract

Molecular profiling of childhood CNS tumors is critical for diagnosis and clinical management, yet tissue access is restricted due to the sensitive tumor location. We developed a targeted deep sequencing platform to detect tumor driver mutations, copy number variations, and heterogeneity in the liquid biome. Here, we present the sensitivity, specificity, and clinical relevance of our minimally invasive platform for tumor mutation profiling in children diagnosed with CNS cancer.

## Introduction

Central nervous system (CNS) tumors are the leading cause of childhood cancer-related death^1^. Molecular classification of tumor subtype is increasingly important for clinical management^1,2^, yet remains a challenge due to sensitive neuroanatomical locations and restricted tissue access. CNS tumors, including diffuse midline glioma (DMG), exhibit mutational heterogeneity^3,4^ which may not be captured by diagnostic surgical biopsy^5^. Moreover, these tumors are not amenable to repeat surgical biopsies, thus tumor genome evolution at disease progression^5^ remains largely unexplored.

To address the lack of ‘tumor visibility’, we previously developed a digital droplet PCR (ddPCR)-based approach to detect DMG driver mutations in circulating tumor DNA (ctDNA) from patient biofluids^6–8^. However, this approach is suitable only for monitoring a limited number of single nucleotide variants (e.g., up to 4 multiplexed mutations), and is not sufficient to detect larger-scale alterations. To achieve comprehensive tumor mutation profiling in the liquid biome, we validated a commercially available, targeted next generation sequencing (NGS) platform covering a panel of 523 cancer-associated genes (TSO500ctDNA^TM^), encompassing all major prognostic and driver mutations associated with DMG^5,9^ and adapted this platform to detect tumorigenic alterations in ctDNA. In this proof-of-concept study, we evaluated platform performance in a cohort of paired plasma (1-2 mL) and CSF (500 µL-1 mL) specimens from children diagnosed with DMG (*n* = 10, Table 1). Paired tumor tissue was analyzed by whole exome (WES) or whole genome sequencing (WGS).

**Table 1:** Patient cohort for analysis.

PID	Diagnosis	Gender	Age	Specimen	Source	Time death-autopsy	Volume (mL)	[DNA] (ng/µL)	Input (ng)	Exons 500X (%)	MEC
760	DIPG	M	9	C	PM	8 h 20 m	1	<LOQ	<LOQ	0.01	54
				P	PM		2	3.1	50	96	2976
765	DIPG	F	3	C	PM	10 h 45 m	1	54	100	70	619
				P	Live, DT		2	0.3	11.6	78	606
846	DIPG	F	7	C	PM	13 h	1	24.5	60	90	1572
				P	Live, DT		2	0.2	8.6	8.4	653
889	DIPG	F	4	C	PM	49 h	1	14.5	100	97	4346
				P	Live, DT		2	0.2	8	56	515
933	DIPG	F	13	C	Live, PT, IO		0.5	305	100	94	2298
				P	Live, PT		2	0.2	7.8	73	559
1279	DIPG	F	NA	C	PM	24 h	1	0.1	4.2	0.03	164
				S	PM		1.5	8.9	50	62	643
1291	DIPG	F	4	C	PM	96 h*	1	55	100	97	3849
				P	Live, PT		1	0.2	6.6	62	273
1368	DIPG	F	7	C	PM	5 h	1	5	100	96	1868
				P	PM		1	2.5	50	94	3674
1446	DIPG	F	6	C	PM	N/A	1	3.1	100	98	4155
				S	PM		2	3.5	50	76	2172
1549	Thalamic DMG	M	9	C	Live		1	<LOQ	<LOQ	0	29
				P	Live		1.5	0.9	36.5	70	609
Median				C				9.8	100	92	1720
				P/S				0.6	24.1	74.5	626

Ten children diagnosed with DMG (*n* = 9 DIPG, 1 thalamic DMG), were included in the study. This table lists patient demographic characteristics (gender, age at diagnosis [years]), and specimen details: biofluid type (*C* CSF, *P* plasma, *S* serum); collection source (*PM* postmortem, *PT* pre-treatment, *DT* during treatment, *IO* intra-operative); time elapsed from death to autopsy processing, for PM specimens (in hours[h] and minutes[m])*; volume for DNA isolation (‘Vol’, mL); DNA concentration [DNA] (ng/µL); DNA input (ng) used to generate sequencing libraries; and quality control metrics of the resulting libraries (Exons 500X, MEC). Bottom row lists the median DNA concentration, starting DNA input, Exons 500X, and MEC for all CSF (C) and plasma/serum (P/S) specimens.

*DMG* diffuse midline glioma, *DIPG* diffuse intrinsic pontine glioma, *<LOQ* below the limit of quantification by Qubit High Sensitivity assay, *Exons 500X* percentage of exons with ≥500X coverage, *MEC* median exon coverage.

*Approximated time from death to autopsy processing for PID 1291 (exact time N/A).

## Results and discussion

### Benchmarking ctDNA sequencing library preparation

**Fig. 1:**
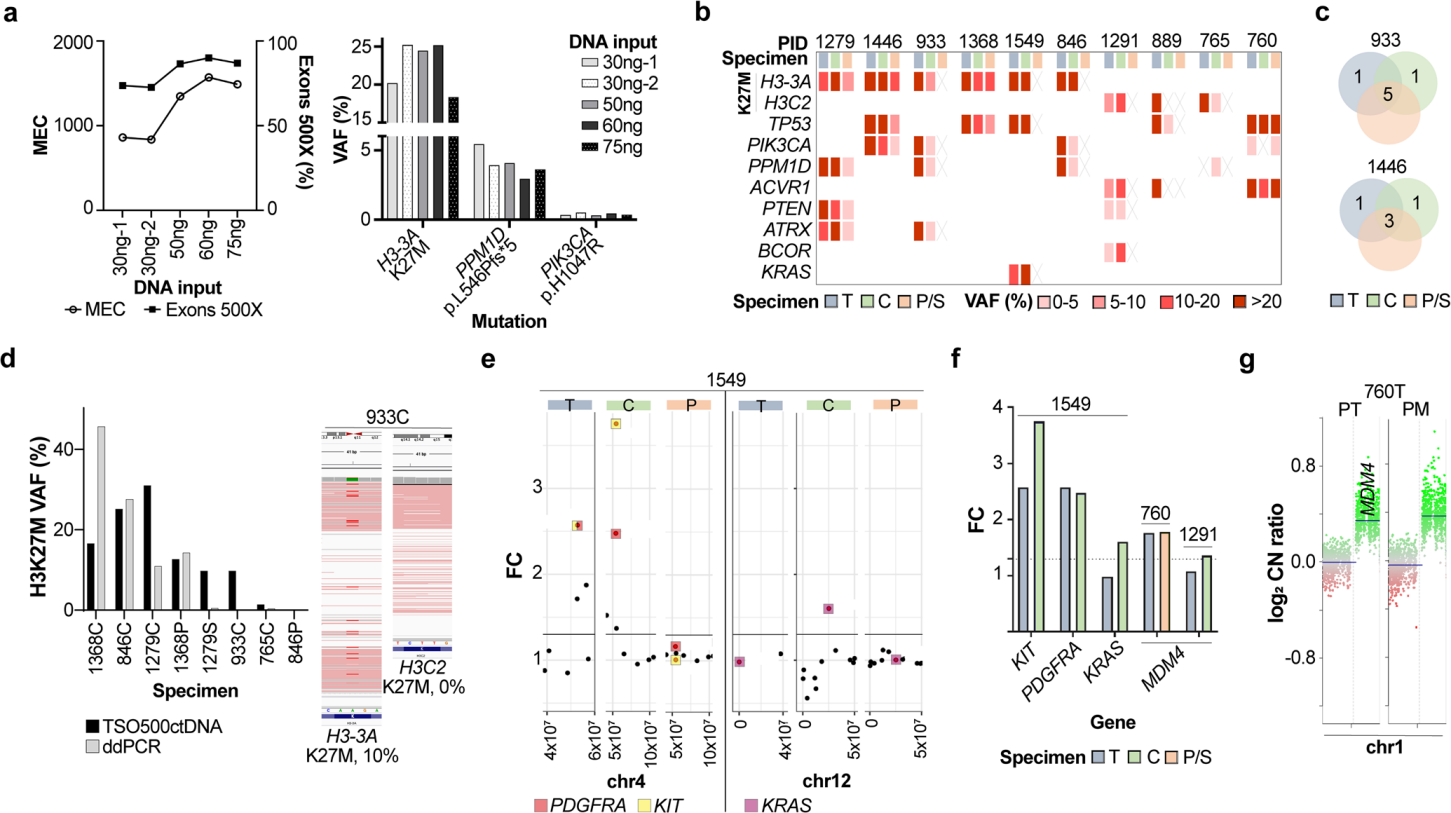
Detection of tumorigenic alterations in paired DMG tissue, CSF, and plasma/serum. **a** Left: MEC (left y-axis) and Exons 500X (%; right y-axis) of sequencing libraries generated from 30–75 ng DNA input (ID 846CSF). ‘30ng-1’ and ‘30ng-2’ indicate technical replicates of 30 ng inputs. Right: Detection of DMG-associated mutations in genes *H3-3A, PPM1D*, and *PIK3CA* from libraries generated at 30, 50, 60, and 75 ng input. **b** Concordance of DMG-associated mutations detected in paired tumor tissue (T), CSF (C), and plasma (P) or serum (S). ‘X’: variant detected in tumor but not in paired liquid specimen. VAF variant allele frequency. **c** Representative overlap between tumor-associated mutations (SNVs, insertion/deletions) identified in paired specimens from two patients (IDs 933 and 1446). **d** Left: Comparison of H3K27M detection by ctDNA sequencing (black) and ddPCR (grey) in paired specimens (*n* = 8). Right: Detection of *H3-3A* K27M (10% VAF) by deep sequencing (ID 933CSF), with zero false positive reads of *H3C2* K27M. **e** CNV plots showing chromosomes (chr) 4 and 12 (ID 1549). *KIT* and *PDGFRA* gains were detected in tumor and CSF, while *KRAS* gain was detected exclusively in CSF. X-axis = chromosome position; y-axis = fold-change (FC) calculated based on pre-established baseline; y = 1.3, CNV reporting threshold. **f** Detection of *KIT* (*n* = 1)*, PDGFRA* (*n* = 1), *KRAS* (*n* = 1) and *MDM4* (*n* = 2) gains in tumor and biofluid pairs. y = 1.3: CNV reporting threshold. **g** DNA methylation array-derived CNV plots of chr1 from pre-treatment (PT) and postmortem (PM) tumor tissue (ID 760), showing gain of chr1q containing *MDM4*, as validation of *MDM4* gain. Y-axis: log-2 CN ratio. T tumor, C CSF, P plasma, S serum, CN copy number.

Sequencing libraries were generated from cell free DNA between 90–250 base pairs (bp) in length to capture tumor-derived fragments^10,11^. We first benchmarked three metrics of ctDNA library preparation: (1) DNA input, (2) percentage of target exons with ≥500X coverage (‘Exons 500X’), and (3) median exon coverage (MEC). We sequenced multiple libraries from CSF (ID 846) ranging from 30–75 ng input (Fig. 1a, Supplementary Data 1). High assay reproducibility was observed when sequencing two replicates of 30 ng input DNA (Fig. 1a). All DNA inputs tested generated high quality libraries, with optimal QC results obtained at 60 ng. Specifically, 30 ng input resulted in Exons 500X of 73–74%, whereas increasing to 60 ng input improved Exons 500X to >90% **(**Fig. 1a, left). DMG driver mutations were detected at all inputs (Fig. 1b, right). Thus, subsequent CSF specimens were analyzed at ≥60 ng input where attainable. Plasma/serum specimens were analyzed at ≥50 ng input, given lower DNA yields (Table 1). Most (*n* = 7/10) CSF specimens achieved target yield (≥60 ng), and all liquid specimens successfully generated sequencing libraries.

### ctDNA deep sequencing reveals clinically relevant mutations

Deep sequencing achieved concordant mutation detection in paired tumor tissue and biofluids (Fig. 1b). In general, tumor and CSF pairs displayed higher concordance than tumor and plasma/serum pairs (Fig. 1b, c). Given that our cohort was comprised of DMGs, we evaluated detection of histone H3K27M mutation, present in 80% of DMGs and most commonly affecting *H3-3A* (60%) or *H3C2* (20%) genes^12–15^. Most patients in our cohort harbored H3K27M (*n* = 9/10), confirmed by tumor DNA sequencing (Fig. 1b). Of the nine H3K27M mutations detected in tumor, eight were present in CSF and three in plasma/serum, for a positive percent agreement of 89% and 33%, respectively, with tumor results. We compared these results to our ddPCR-based H3K27M assay^6,7^ using eight paired liquid specimens, and detected similar H3K27M variant allele frequencies (VAF) using both approaches (Fig. 1d). In one case (ID 933CSF), ddPCR results were inconclusive, with one false positive *H3C2* K27M-mutant allele detected (Supplementary Data 2). In contrast, deep sequencing resolved histone mutation status, detecting *H3-3A* K27M (9.8% VAF) with zero false positive reads at *H3C2* locus, in agreement with tumor sequencing results (Fig. 1d). In one patient wildtype for H3 (ID 760), zero false positive reads of H3K27M were detected. Collectively, our results demonstrated high platform sensitivity and specificity for CSF mutation detection.

We next profiled clinically relevant histone partner mutations^9,15^ and detected alterations in genes including *TP53, PPM1D*, and *PTEN* in tumor and ctDNA pairs (Fig. 1b). To evaluate the clinical utility of our platform, we focused on CSF collected at upfront diagnosis or during the course of treatment (IDs 933, 1549). In these live CSF specimens, we identified several tumor driver mutations including *H3-3A*, *TP53, PPM1D*, *ATRX*, and *KRAS* mutations, which were confirmed present in paired tumor (Fig. 1b). Strikingly, although 1549 CSF yielded immeasurably low DNA (Table 1), oncogenic mutations were detected including H3K27M, *KRAS* and *TP53* gain-of-function mutations (44-53% VAF) (Fig. 1b), supporting the utility of our platform for early detection of clinically relevant mutations even in cases with extremely low starting DNA input.

### Capturing tumor genomic heterogeneity in liquid biome

We next assessed whether ctDNA was informative of tumor heterogeneity when compared to tissue. In one case (ID 1291CSF) we identified mutations including *PTEN* deletion, *BCOR* frameshift, and *ACVR1* activating mutation that were absent from tumor WES. In a second case (ID 760 P), we detected a low frequency *PIK3CA* activating mutation, also absent from paired tumor WES. We inspected WES data and found that these variants were indeed present in raw sequencing files at subthreshold VAF (<10%), thus filtered and not reported. In line with our previous work^5,9^, mutations uncovered by liquid biome analyses may represent low frequency/sub-clonal events, further emphasizing the temporal and spatial heterogeneity and sub-clonal evolution of the DMG genome. Our observation indicates a potential advantage of ctDNA analyses to uncover tumor tissue genomic heterogeneity, including detection of sub-clonal mutations activating key DMG-associated growth factor signaling pathways.

### Detection of tumor copy number variation in ctDNA

Finally, we assessed the feasibility of tumor copy number variation (CNV) detection in ctDNA. Interestingly, our platform proved more informative for CNV detection when compared to tumor WES. In patient 1549, CSF and tumor specimens showed concordance on *KIT* and *PDGFRA* gains (Fig. 1e, f). However, *KRAS* gain was detected exclusively in CSF, albeit at a relatively modest amplification (<2-fold change), representing a putative sub-clonal event not captured by tumor WES. Notably, this patient also harbored *KRAS* activating mutation, suggesting that *KRAS* gain may be associated with oncogene activation in this tumor, as reported in other cancers^16–18^. Additionally, *MDM4* gains were detected in ctDNA (IDs 760, 1291) (Fig. 1f). In patient 760, tumor WES indicated a sub-threshold, but similar, gain of *MDM4* (fold change 1.76 and 1.78 in tumor and plasma, respectively). This copy number gain, detected at a similar level by tumor and ctDNA sequencing, was subsequently confirmed by tumor DNA methylation analyses revealing amplification of chromosome 1q harboring *MDM4* locus (Fig. 1g). Further studies are warranted to optimize the appropriate thresholds for CNV detection in ctDNA using large cohorts of CNS cancer and healthy pediatric control CSF and plasma.

## Discussion

Our collective data present a minimally invasive, targeted deep sequencing platform capable of detecting and monitoring clinically relevant mutations in the liquid biome of children diagnosed with DMG and other CNS tumors, with remarkable sensitivity and specificity in CSF specimens. While CSF collection for molecular analyses is routine for children and young adults with certain CNS cancers (e.g., medulloblastoma and other embryonal tumors^19^), the procedure is currently not part of standard-of-care for children diagnosed with DMG. Our findings provide an opportunity for prospective clinical trials to incorporate CSF collection and deep sequencing for DMG molecular profiling as a less invasive alternative to surgical tissue biopsies. Though technology is becoming increasingly sensitive, future studies are needed to improve somatic mutation detection in plasma/serum. Additionally, one limitation of our study was the inclusion of retrospectively collected and postmortem specimens. Though the time from death to autopsy processing (Table 1) did not correlate to the total cell free DNA concentration, nor to the maximum somatic VAF detected in CSF (Supplementary Fig. 1), further studies are warranted using larger cohorts of live CSF specimens. These prospective studies will require standardization of collection time and processing protocols for uniformity across specimens. Importantly, while monitoring changes in the frequency of single hotspot mutations in ctDNA provides only limited insight into treatment response in DMGs^7,20^, the use of a large gene panel provides a comprehensive profile of the evolving tumor genomic landscape. Our results establish a foundation for comprehensive tumor mutation profiling in ctDNA, improving the ability to assess tumor molecular heterogeneity and genomic evolution in children diagnosed with CNS cancer.

## Methods

### Biological specimens

All patient specimens were collected after written informed consent was obtained from each patient or patient’s guardian for participation in a clinical trial, or biorepository as approved by the respective Institutional Review Boards of Children’s National Hospital (IRB #1339, #747) and University of California San Francisco (San Francisco, CA; IRB #14-13895). Patient specimens were collected during treatment (PNOC003, NCT02274987) or upon autopsy. All patient identifiers were removed with de-identified numerical identifiers were assigned. CSF from children with DMG (*n* = 10, Table 1) was collected during treatment or at autopsy. CSF specimens were centrifuged for 5000 *×* *g* for 10 min at 4 °C, supernatant was collected, aliquoted and stored at −80 °C. Plasma (*n* = 8) from children with DMG was collected as whole blood in purple top potassium EDTA tubes, inverted, and centrifuged at 2000 *×* *g* for 15 min at 4 °C. Serum (*n* = 2) from children with DMG was collected in gel-barrier tubes with clot activator and gel, incubated for 30 min at room temperature, and centrifuged at 2000 *×* *g* for 15 min at 4 °C. Plasma/serum supernatants were aliquoted into cryovials and stored at −80 °C. Tumor tissue (*n* = 10) was obtained from DMG patients during treatment or at autopsy and stored at −80 °C.

### DNA isolation and quantification

Tumor tissue genomic DNA was isolated from frozen tissue specimens using Gentra Puregene Tissue Kit (QIAGEN) per the manufacturer’s instructions. Genomic DNA was quantified using Qubit dsDNA Broad Range Assay Kit. Cell free DNA was isolated from 500 µL-1 mL of CSF and 1-2 mL of plasma or serum (Table 1) using QIAamp Circulating Nucleic Acid Kit (QIAGEN) (ID 933CSF) or MagMax Cell-Free DNA Isolation Kit (ThermoFisher) (all other liquid specimens) according to the manufacturers’ instructions. DNA quantity was assessed using Qubit dsDNA High Sensitivity Assay kit.

### Tape Station analysis of cell free DNA

Cell free DNA size was evaluated using the Agilent Technologies TapeStation 4200 High Sensitivity D5000 assay according to the manufacturer’s instructions.

### Digital droplet PCR

ddPCR detection and quantification of *H3-3A* and *H3C2* K27M variant allelic frequency (VAF) were performed as previously described^6,7^. Briefly, cell free DNA isolated from 500 µL CSF or 1 mL plasma/serum was subjected to pre-amplification using sequence-specific primers, dropletization, and PCR using sequence-specific primers and fluorescent probes. The numbers of mutant and wildtype droplets were quantified using RainDrop Analyst II Software (RainDance Technologies). VAF values were calculated as the number of mutant droplets divided by the sum of mutant and wildtype droplets. H3K27M VAF values represent the average of technical duplicates (CSF) or triplicates (plasma/serum).

### Tumor whole exome sequencing

Whole exome libraries from each sample were generated by shearing 50 ng of genomic DNA to 150–180 bp using Covaris LE220 sonicator (Covaris, Woburn, MA). The following library preparation procedure was automated on a SciClone G3 liquid handling workstation using custom scripts. Sheared genomic DNA was processed using Kapa Hyper library construction and dual index kit (Kapa/Roche, Wilmington, MA) through end-repair, dA tailing, ligation with indexed Illumina adaptors on a SciClone G3 liquid handling workstation (Perkin Elmer, Waltham, MA). Adaptor-ligated libraries were purified using AMPure XP beads (Beckman Coulter, Indianapolis, IN) and then amplified by PCR (12 for germline specimens or 14 cycles for tumor FFPE specimens) using KAPA HiFi polymerase (KAPA/Roche). Amplified libraries were purified using AMPure XP beads, and 750 ng of each library was hybridized to a biotinylated RNA bait set (SureSelect XT Human V6 + COSMIC, Agilent Technologies, Santa Clara, CA) at 65 °C for 16 h. The captured genomic DNA fragments were enriched by Dynabeads MyOne Streptavidin T1 beads (Invitrogen/ThermoFisher Scientific) and amplified for 10 cycles of PCR using KAPA HiFi polymerase (KAPA/Roche). Amplified post-hybridization libraries were purified using AMPure XP beads, checked for size distribution (300–400 bp) using Agilent TapeStation 4200 and quantitated using Quant-iT™ High-Sensitivity dsDNA assay kit (ThermoFisher Scientific) on a SpectraMax M2e microplate reader (Molecular Devices, Hampton, NH). Final libraries were pooled at equimolar ratios and quantitated with a ddPCR Library Quantification Kit for Illumina TruSeq (Bio-Rad) on a QX200 digital PCR system (BioRad Laboratories, Hercules, CA). Final pooled libraries were diluted to either 1.2 nM or 2.0 nM depending on NovaSeq run mode: XP or Standard. Then, denatured final pool was clustered on a NovaSeq S4 flowcell (Illumina, San Diego, CA). Sequencing was performed on a NovaSeq 6000 (Illumina) using 2 × 150 bp paired-end sequencing mode.

### Analysis of tumor-only WES data

An in-house bioinformatics pipeline was used to process tumor-only WES data. FASTQ data were generated using the bcl2fastq tool (Illumina, v2.18) and run through FASTQC for quality confirmation. Reads were mapped to the human hg19 reference genome using the Burrows-Wheeler alignment tool^21^. The resulting bam files were processed using GATK best practice workflow^22^. GATK HaplotypeCaller and Platypus^23^ were used to call variants. Copy number data was inferred from WES data through use of the CNVKit algorithm^24^, using a pool of normal Hapmap cell line specimens as references. The variants identified by WES were further annotated by the MoCha Oncogenic MOI Annotator (MOMA: https://github.com/FNL-MoCha/moma), a sequencing platform agnostic tool used to annotate variants as Mutations of Interest (MOIs) or Variants of Unknown Significance (VuS). These classifications are based on data from annotating variants with Annovar^25^ and mapping variants to OncoKB^26^.

### Targeted cell free DNA NGS

Libraries were prepared using ≤50 ng DNA originating from plasma and serum, and ≤100 ng from CSF, using the Illumina TruSight^TM^ Oncology 500 ctDNA with unique molecular identifiers (UMIs) and duplex barcodes for error correction, then enriched by target capture, pooled, and denatured according to the manufacturer instructions, and as described^27^. The denatured final pool was clustered on a NovaSeq S4 flowcell (Illumina, San Diego, CA). Sequencing was performed on a NovaSeq 6000 (Illumina) using 2 × 150 bp paired-end sequencing in XP mode.

### TSO500ctDNA sequencing data analysis

Cell free DNA sequencing data were analyzed using the TSO500 analytical pipeline zipmocom 0.8.2.10, as described in Zhao et al.^27^. The TSO500ctDNA analysis pipeline was modified from the original FFPE pipeline^27^. This pipeline included Burrows-Wheeler Aligner to align reads to the reference genome; Unique Molecular Identifiers (UMIs) for read collapsing; Gemini to stitch together collapsed sequences into consensus fragments; Pisces somatic variant calling; and Nirvana to annotate variants. The variants annotated by this analysis were further annotated by MOMA as described above.

The original FFPE pipeline was modified to allow for lower VAF detection, and used a baseline derived from healthy donor plasma specimens to subtract background signal specific to cell free DNA materials. Alignment, collapsing, utilization of UMIs and post hoc filtering for germline variants were shared between the two pipelines, using thresholds relevant for different sample types. Limits of detection were set as 0.25% VAF for single nucleotide variants and insertion/deletions, 0.5% for translocations, and 1.3-fold change for CNVs.

### Analysis of 765T whole genome sequencing data

One tumor tissue sample (765T) failed to generate a WES library. Thus, we queried previously generated, publicly available WGS data from this sample to determine tumor mutation status. Data were available from the Children’s Brain Tumor Network (CBTN) on the Cavatica platform (cavatica.sbgenomics.com).

To estimate VAF of mutations of interest in 765T, the somatic mutation caller Mutect2 in tumor-only mode from GATK^28^ was used.

### DNA methylation analysis

DNA was extracted from frozen tumor tissue (ID 760), bisulphite converted using EZ DNA Methylation-Gold kit (Zymo Research) and hybridized onto Infinium MethylationEPIC BeadChip using Infinium MethylationEPIC BeadChip Kit per the manufacturer’s instructions (Illumina). BeadChip arrays were scanned using iScan Reader (Illumina). Data (IDAT files) were uploaded to the Molecular Neuropathology CNS Tumor Classifier Version v11b4 (https://www.molecularneuropathology.org/mnp) to generate CNV plots^29^.

### Primer sequences

For ddPCR detection of H3K27M mutation, *H3-3A* and *H3C2* primer sequences are listed in Panditharatna et al.^7^. For DNA sequencing, standard Illumina sequencing primers were used, are sequences are available at: https://support-docs.illumina.com/SHARE/AdapterSeq/Content/SHARE/AdapterSeq/TruSight/Tumor170/TruSightTumor500ctDNA.htm.

### Statistical analysis

To test for correlations between the time from death to autopsy processing and (a) CSF cell free DNA concentration or (b) maximum somatic VAF detected in CSF (Supplementary Fig. 1), Spearman correlations were performed and two-tailed p-values were reported.

### Reporting summary

Further information on research design is available in the Nature Research Reporting Summary linked to this article.

## Supplementary information

Supplementary Information

Reporting Summary

Supplementary Data 1. Evaluation of assay reproducibility and feasibility.

Supplementary Data 2. Comparison of ddPCR and deep sequencing for H3K27M detection in paired liquid specimens.

## Data Availability

All sequencing datasets generated during the current study are available from the Blood Profiling Atlas in Cancer (BloodPAC) Data Commons (dataset identifier bpa-NCI). The sequencing data presented in the current publication have also been deposited in and are available from the dbGaP database under dbGaP accession phs003022.v1.p1. DNA methylation array data discussed in this publication have been deposited in NCBI’s Gene Expression Omnibus and are accessible through GEO Series accession number GSE210323 (https://www.ncbi.nlm.nih.gov/geo/query/acc.cgi?acc=GSE210323).

## Code availability

Code used to analyze sequencing data are available at: https://github.com/FNL-MoCha/nextgenseq_pipeline.
