# Supplementary material for: Circulating tumor DNA sequencing provides comprehensive mutation profiling for pediatric central nervous system tumors
Source: NPJ Precis Oncol. 2022 Sep 6;6:63. doi: 10.1038/s41698-022-00306-3 (PMC9448784; doi:10.1038/s41698-022-00306-3)
Supplement: Supplementary file 1 — Supplementary Information [file 41698_2022_306_MOESM1_ESM.pdf]

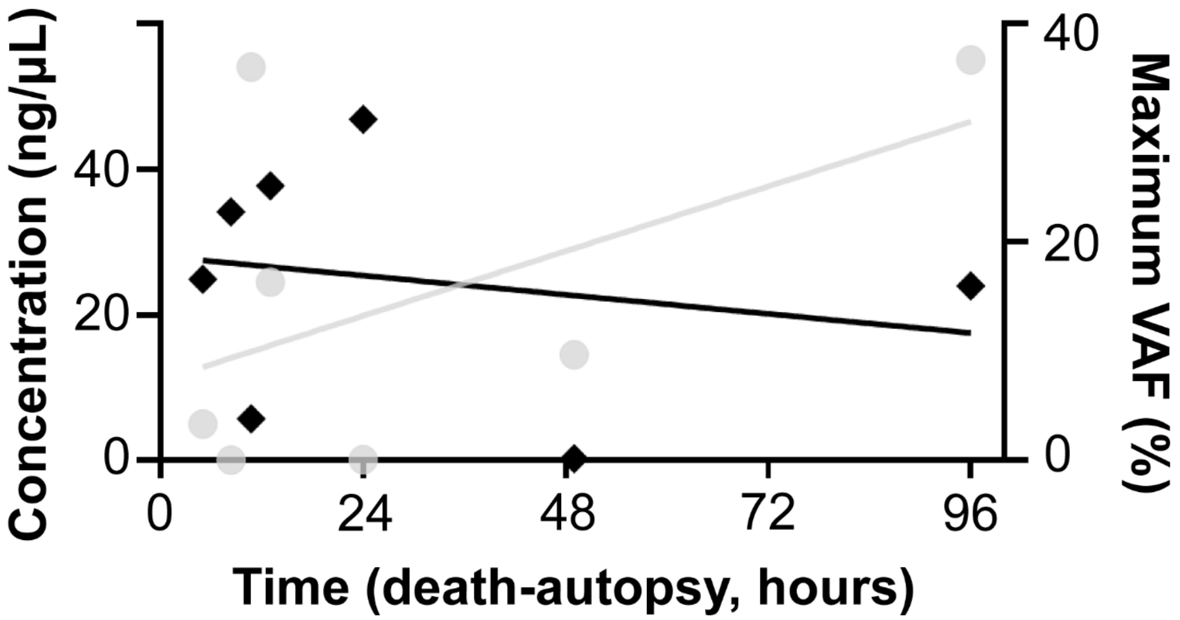

Variable	N	P value	Summary
Cell free DNA concentration	7	0.2667	n.s.
Maximum VAF	7	0.6615	n.s.

Supplementary Figure 1. **Postmortem processing time did not correlate to CSF cell free DNA concentration or tumor mutation detection.** The time from death to autopsy processing (hours, x-axis) did not correlate to total cell free DNA concentration (ng/µL, left y-axis) or to maximum tumor VAF detected in CSF (%, right y-axis). Spearman correlations with two-tailed p-values are represented. N=7 CSF samples. *Abbreviations:* CSF=cerebrospinal fluid; VAF=variant allele frequency.

Supplementary Data Legends

Supplementary Data 1. Evaluation of assay reproducibility and feasibility. To evaluate assay reproducibility, two technical replicates of 30ng CSF DNA (ID 846) were sequenced. Sheet one (“1.QC”) lists sequencing library quality control (QC) metrics, including library conversion efficiency (%), DNA yield (ng/µL), median insert size (median fragment length of the library), mean family depth, uniformity 20% (percentage of target bases with fragment coverage >0.2 times the mean region target coverage),

median exon coverage (MEC), percentage of exons with 500x coverage (Exons 500X), median of absolute deviations (MAD), and median bin count.

To benchmark cell free DNA input for optimum library generation, DNA inputs of 30ng, 50ng, 60ng, and 75ng were sequenced and results were compared. For each starting DNA input, the first sheet ("1.QC") lists QC metrics, and the second sheet ("2.Compiled_Results") lists the variants above the limit of reporting in at least one replicate. These variants include *H3-3A*, *PAX5*, *PIK3CA*, *PPM1D*, and *SLX4* mutations. Beneath these variants are listed variants below the limit of reporting in all reported replicates. The third sheet ("3.846CSF_Complete_Output") contains the full variant call output for each library. DMG genes of interest (*H3-3A*, *PIK3CA*, *PPM1D*) are highlighted in green.

Supplementary Data 2. Comparison of ddPCR and deep sequencing for H3K27M detection in paired liquid specimens. Results for H3K27M mutation detection (VAF) are listed for each CSF and plasma/serum specimen analyzed by ddPCR and by TSO500ctDNA deep sequencing. N/A: the sample was not profiled by the indicated platform. *Abbreviations:* ddPCR=digital droplet polymerase chain reaction; VAF=variant allele frequency; CSF=cerebrospinal fluid.
