# Supplementary material for: Circulating tumor DNA sequencing provides comprehensive mutation profiling for pediatric central nervous system tumors
Source: NPJ Precis Oncol. 2022 Sep 6;6:63. doi: 10.1038/s41698-022-00306-3 (PMC9448784; doi:10.1038/s41698-022-00306-3)
Supplement: Supplementary file 2 — Reporting Summary [file 41698_2022_306_MOESM2_ESM.pdf]

nature portfolio

Corresponding author(s): Javad Nazarian

Last updated by author(s): Feb 28, 2022

Reporting Summary

Nature Portfolio wishes to improve the reproducibility of the work that we publish. This form provides structure for consistency and transparency in reporting. For further information on Nature Portfolio policies, see our Editorial Policies and the Editorial Policy Checklist.

Statistics

For all statistical analyses, confirm that the following items are present in the figure legend, table legend, main text, or Methods section.

n/a	Confirmed	
☐	☒	The exact sample size (*n*) for each experimental group/condition, given as a discrete number and unit of measurement
☒	☐	A statement on whether measurements were taken from distinct samples or whether the same sample was measured repeatedly
☐	☒	The statistical test(s) used AND whether they are one- or two-sided<br>*Only common tests should be described solely by name; describe more complex techniques in the Methods section.*
☒	☐	A description of all covariates tested
☒	☐	A description of any assumptions or corrections, such as tests of normality and adjustment for multiple comparisons
☒	☐	A full description of the statistical parameters including central tendency (e.g. means) or other basic estimates (e.g. regression coefficient) AND variation (e.g. standard deviation) or associated estimates of uncertainty (e.g. confidence intervals)
☒	☐	For null hypothesis testing, the test statistic (e.g. *F*, *t*, *r*) with confidence intervals, effect sizes, degrees of freedom and *P* value noted<br>*Give P values as exact values whenever suitable.*
☒	☐	For Bayesian analysis, information on the choice of priors and Markov chain Monte Carlo settings
☒	☐	For hierarchical and complex designs, identification of the appropriate level for tests and full reporting of outcomes
☒	☐	Estimates of effect sizes (e.g. Cohen's *d*, Pearson's *r*), indicating how they were calculated

Our web collection on statistics for biologists contains articles on many of the points above.

Software and code

Policy information about availability of computer code

Data collection	No software was used for data collection in this study.

Data analysis

Digital droplet PCR data were analyzed using RainDrop Analyst II Software (RainDance Technologies).
Tumor-only WES data were analysed as follows: FASTQ data were generated using bcl2fast1 tool (Illumina v2.18) and run through FASTQC for quality confirmation. Reads were mapped to human hg19 reference genome using Burrows-Wheeler alignment tool. Resulting bam files were processed using GATK best practice workflow. GATK HaplotypeCaller and Platypus were used to call variants. Copy number data was inferred from WES data through use of CNVKit algorithm, using a pool of normal Hapmap cell line samples as references. Variants identified by WES were annotated by MoCha Oncogenic MOI Annotator (MOMA: https://github.com/FNL-MoCha/moma). Classifications as Mutations of Interest (MOIs) or Variants of Unknown Significance (VuS) were based on data from annotating variants with Annovar and mapping variants to OncoKB.
Cell free DNA sequencing data were analyzed using TSO500 analytical pipeline as described in Zhao et al. (2020) TruSight Oncology 500: Enabling Comprehensive Genomic Profiling and Biomarker Reporting with Targeted Sequencing. BioRxiv, doi: 10.1101/202.10.21.349100. This pipeline included Burrows-Wheeler Aligner to align reads to the reference genome; Unique Molecular Identifiers for read collapsing; Gemini to stitch together collapsed sequences into consensus fragments; Pisces somatic variant calling; and Nirvana to annotate variants. Variants were further annotated by MOMA as described above for tumor-only WES variants.
Whole genome sequencing data for one tumor specimen (ID 765T) were downloaded from the Children's Brain Tumor Network (CBTN) on the Cavatica platform (cavatica.sbgenomics.com) and variants of interest were screened for using the somatic mutation caller Mutect2 in tumor-only mode from GATK.
DNA methylation array data were analyzed by uploading IDAT files to the Molecular Neuropathology CNS Tumor Classifier Version 11b4 (https://www.molecularneuropathology.org/mnp) to generate copy number variation plots.
Spearman correlations were used to test for correlations between time from death to autopsy processing and (a) total cell free DNA concentration or (b) maximum tumor variant allele frequency detected in CSF samples. Sample sizes and two-tailed p-values were reported in the corresponding Supplementary Figure (1).

For manuscripts utilizing custom algorithms or software that are central to the research but not yet described in published literature, software must be made available to editors and reviewers. We strongly encourage code deposition in a community repository (e.g. GitHub). See the Nature Portfolio guidelines for submitting code & software for further information.

Data

Policy information about availability of data

All manuscripts must include a data availability statement. This statement should provide the following information, where applicable:

- Accession codes, unique identifiers, or web links for publicly available datasets
- A description of any restrictions on data availability
- For clinical datasets or third party data, please ensure that the statement adheres to our policy

All datasets generated during and analyzed in the current study will be deposited and available according to the journal regulations.

Field-specific reporting

Please select the one below that is the best fit for your research. If you are not sure, read the appropriate sections before making your selection.

☒ Life sciences ☐ Behavioural & social sciences ☐ Ecological, evolutionary & environmental sciences

For a reference copy of the document with all sections, see nature.com/documents/nr-reporting-summary-flat.pdf

Life sciences study design

All studies must disclose on these points even when the disclosure is negative.

Sample size

Paired tumor, cerebrospinal fluid, and blood from 10 patients diagnosed with diffuse midline glioma were used for a proof-of-concept/pilot study to evaluate the feasibility of sequencing circulating tumor DNA to detect tumor mutations in patient biofluids.

Data exclusions

No data were excluded from the analysis.

Replication

Multiple next generation sequencing libraries from the same biospecimen were generated in order to evaluate assay reproducibility/replicability.

Randomization

There were no experimental groups used in this study and therefore no need for randomization of groups.

Blinding

Blinding was not relevant to the study because there were no randomized experimental groups assigned in the study.

Reporting for specific materials, systems and methods

We require information from authors about some types of materials, experimental systems and methods used in many studies. Here, indicate whether each material, system or method listed is relevant to your study. If you are not sure if a list item applies to your research, read the appropriate section before selecting a response.

Materials & experimental systems

n/a	Involved in the study	
☒	☐	Antibodies
☒	☐	Eukaryotic cell lines
☒	☐	Palaeontology and archaeology
☒	☐	Animals and other organisms
☐	☒	Human research participants
☒	☐	Clinical data
☒	☐	Dual use research of concern

Methods

n/a	Involved in the study	
☒	☐	ChIP-seq
☒	☐	Flow cytometry
☒	☐	MRI-based neuroimaging

Human research participants

Policy information about studies involving human research participants

Population characteristics	Age, gender, and patient diagnosis were included in the study.
Recruitment	Patient samples were obtained after written informed consent from patients, or patient guardians, enrolled in a clinical trial or biorepository as approved by the respective Institutional Review Boards.
Ethics oversight	Institutional Review Boards of Children's National Hospital (IRB #1339, #747) and University of California San Francisco (San Francisco, CA; IRB #14-13895).

Note that full information on the approval of the study protocol must also be provided in the manuscript.
